# The impact of sedentarism on heart rate variability (HRV) at rest and in response to mental stress in young women

**DOI:** 10.14814/phy2.13873

**Published:** 2018-09-20

**Authors:** José Robertto Zaffalon Júnior, Ariane Oliveira Viana, Gileno Edu Lameira de Melo, Kátia De Angelis

**Affiliations:** ^1^ State of Pará University (UEPA) Altamira Pará Brazil; ^2^ Nove de Julho University (UNINOVE) São Paulo São Paulo Brazil; ^3^ Federal University of São Paulo (UNIFESP) São Paulo São Paulo Brazil

**Keywords:** Heart rate variability, lifestyle, quality of life, women

## Abstract

Sedentarism is one of the main risk factors for the onset of cardiometabolic diseases. Some biomarkers, such as heart rate variability (HRV), have been largely studied and found to be involved in the genesis of the dysfunctions associated with sedentary behavior. However, comparatively few studies have focused on the female sex. The objective of this study was to analyze the hemodynamic, autonomic and quality of life parameters at rest and in response to mental stress of sedentary and physically active young women. A total of 96 women, 18–30 years of age, were divided into sedentary (SW = 48) and active (AW = 48) groups. Anthropometric, hemodynamic and quality of life parameters were evaluated and the R‐R interval was recorded to quantify the cardiac autonomic modulation at rest and in response to the Stroop Color Test. The groups were similar in age, weight, height, body mass index, fat percentage, systolic and diastolic blood pressure values and glycemia. The physical health domain of quality of life was compromised in the SW group. The SW group presented higher heart rate, lower variance of RR interval and RMSSD and higher cardiac sympathovagal balance (LF/HF) both at rest and in response to the mental stress test. We concluded that sedentary lifestyle in women induces impairment in autonomic cardiac modulation at rest and in response to physiological stress, compromising the quality of life, even before altering any cardiovascular or metabolic clinical parameters, reinforcing the potential role of HRV as early marker of cardiovascular risk in this population.

## Introduction

Sedentary behavior has recently emerged as a strong risk factor for cardiometabolic diseases. Several studies have shown that this behavior is associated with increased body weight and blood pressure, as well as with unfavorable changes in the lipid and glycemic profile (Marques et al. 2018). On the other hand, regular exercise can induce benefits such as increased aerobic power, strength, muscle mass, flexibility, along with decreased body fat, decreased anxiety, lower stress levels, and blood pressure (Bompa and Buzzichelli 1999).

Cardiometabolic changes resulting from a sedentary lifestyle may take many years to be clinically detectable. For this reason, other biomarkers involved in the genesis of these dysfunctions have been more largely studied. In this sense, heart rate variability is a noninvasive method to assess cardiac autonomic modulation. Changes in HRV patterns serve as health indicators, since a number of studies have suggested that higher HRV values are associated with a positive health status, whereas reduced HRV indicates poor or inadequate adaptation of the autonomic nervous system (ANS), and is associated with increased cardiovascular risk and mortality (Marques et al. 2018). Several factors may affect HRV, such as overweight/obesity, nicotine intake, alcohol, caffeine, vigorous physical exercises, age, gender, posture, amongst others (Valentini and Parati 2009). Additionally, some researchers have pointed to the critical role of the autonomic nervous system in the genesis of cardiovascular disease (Sloan et al. 2007; Tracey 2014).

Many studies have demonstrated that physical activity increases HRV, but they have largely focused on assessing autonomic modulation among male athletes of different sports modalities (Vesterinen et al. 2013), physically active and inactive individuals (Mourot et al. 2004) or sedentary subjects exposed to an intensive training program (Iwasaki et al. 2003). Such studies do not separate the autonomic behavior of physically active and inactive women from their male counterparts, which would be extremely important to understand the impact of lifestyle on female cardiovascular health. Ovarian hormones play a key role in the maintenance of low sympathetic modulation (Tenan et al. 2014) and as women age, they present a greater increase in sympathetic modulation than men, which may significantly contribute to systemic arterial hypertension and other cardiovascular diseases (Narkiewicz et al. 2005; Konicki 2012).

It should be remarked that early physiological changes may not be observed in a resting situation, being perceived only when the individual undergoes a stressful situation. In this sense, studies have increasingly applied mental or physical stress tests to promote physiological challenges that may exacerbate abnormal responses in healthy subjects or those affected by diseases (Weidner et al. 2001; Boutcher and Boutcher 2006; Wei et al. 2016). However, we do not know of any studies comparing hemodynamic responses and HRV among sedentary and physically active women after a mental stress test.

The present work is based on the hypothesis that sedentarism may impair HRV in young women at rest and in response to a mental stress test, thus impacting negatively their quality of life. Given that, the aim of this study was to analyze the hemodynamic, autonomic and quality of life parameters at rest and in response to the mental stress in sedentary and physically active young women.

## Materials and Methods

A cross‐sectional study was conducted with 96 women aged 18–30 years old, divided into 2 groups and using the International Physical Activity Questionnaire (IPAQ): sedentary women (SW), *n* = 48, classified as sedentary, irregularly active A or B; active women (AW), *n* = 48, classified as active or very active.

Exclusion criteria were as such: women with diseases which may cause secondary hypertension; women using drugs or any substances which may raise blood pressure; women with a body mass index (BMI) above 30 kg/m^2^; and women with irregular menstrual cycle and pregnant women.

The study participants were informed about the procedures and signed a free and informed consent form. The present study was approved by the Research Ethics Committee with Human Beings of the University of Nove de Julho (UNINOVE) CAAE n° 56952316.1.0000.5511. The procedures are in accordance with the Helsinki Declaration of 1975, as revised in 2008. Participants were instructed to avoid moderate or vigorous physical activity, and to refrain from consuming caffeine, chocolate, nicotine, alcohol, and any other stimulating substances for at least 24 h before the evaluation day. Hemodynamic, autonomic, and glycemic parameters were all measured during either the follicular phase of the menstrual cycle or the phase of high hormonal concentration in women using contraceptive (Rebelo et al. 2012). The percentage of fat, along with weight, height, glycemic, blood pressure, quality of life (SF‐36) and perceived stress level (PSS‐10) were assessed. The psychological stress was evaluated by the Perceived Stress Scale (PSS‐10) instrument (da Silva et al. 2015). In addition to providing a subjective assessment of stress, it highlights the brevity of the instrument, which favors its application in conjunction with other measures (Machado et al. 2014). In summary, the questionnaire quantifies the level of stress perceived by the individual in the face of stressful situations. The instrument consists of 10 questions with 0 to 4 scores and the perceived stress is greater as the score increases.

A validated Brazilian version of the SF‐36 was used to evaluate HRQoL (Ciconelli et al. 1999). The SF‐36 assesses several domains of health‐related HRQoL, including physical functioning and general health perceptions.

The assessment of cardiac autonomic modulation was performed by recording the RR interval using V800 model of the Polar^®^ heart rate monitor. The RR interval was recorded: (1) for a period of 15 min with the subjects at rest; (2) for a period of 15 min after the mental stress test. The HRV was analyzed in three segments of RR recording at rest and in two moments of the mental stress test recovery, called 1st moment: 2–5 min after the mental stress test; and 2nd moment: 6–9 min after the mental stress test.

The spectrum resulting from the Fast Fourier Transforms (FFT) modeling is derived from all the data present in the recorded signal; it includes the entire signal variance, regardless of whether its frequency components appear as specific spectral peaks or as nonpeak broadband powers (Malliani et al. 1991). RR interval (RR) variability was evaluated in the time and frequency domains. The RMSSD (root mean square of the successive differences of the RR interval) reflects the variability in the change in NN interval. Spectral power for low (LF: 0.03–0.15 Hz) and high (HF: 0.15–0.4 Hz) frequency bands, representatives of cardiac sympathetic and parasympathetic modulation, respectively, was calculated by means of power spectrum density integration within each frequency bandwidth, using a customized routine (Cardioseries) (Malliani et al. 1991; da Silva Soares et al. 2004).

The Stroop Color and Word Test (SCWT) is a neuropsychological test used as a mental stress test. It assesses the ability to inhibit cognitive interference, which occurs when the processing of a stimulus feature affects the simultaneous processing of another attribute of the same stimulus. There are three version. In the third version, named color‐word condition, color‐words are printed in an inconsistent color ink (for instance the word “red” is printed in green ink). Thus, in this incongruent condition, the participants are required to perform a less automated task (i.e., naming ink color) while inhibiting the interference arising from a more automated task. This diffculty in inhibiting the more automated process is called the Stroop effect (Scarpina and Tagini 2017).

The Shapiro–Wilk test was used to determine the normal distribution. The results are presented as mean ± standard deviation of the mean, and they were then compared using the Student's *t*‐test of unpaired samples and GraphPad Prism 6 software. The significance level adopted was *P* < 0.05.

## Results

As shown in Table 1, age, body weight, height, body mass index (BMI), fat percentage, systolic (SBP) and diastolic blood pressure (DBP), and glycemia were similar among groups, demonstrating the homogeneity of the sample and allowing the classification of the subjects into normotensive individuals. The AW group presented a significant reduction in basal heart rate when compared to the SW group (Table 1).

**Table 1:** Characterization of the sample, hemodynamic parameters and glycemia in the studied groups

Variables	SW (*n* = 48)	AW (*n* = 48)	*P*
Age (years)	23.1 ± 3.9	23.3 ± 3.7	0.7972
Weight (kg)	56.6 ± 9.3	57.2 ± 9.3	0.7527
Height (m)	1.6 ± 0.1	1.6 ± 0.1	>0.9999
BMI (kg/m^2^)	21.9 ± 3.3	22.4 ± 3.2	0.4530
% Fat	24.3 ± 5.4	23.6 ± 5.2	0.5193
SBP (mmHg)	109.9 ± 12.1	112.1 ± 11.3	0.3596
DBP (mmHg)	70.7 ± 10.2	71.2 ± 9.6	0.8052
HR (bpm)	76.7 ± 10.4	68.8 ± 9.6^a^	**0.0002**
Glycemia (mg/dL)	93.8 ± 12.5	92.5 ± 10.6	0.6623

a Versus. **SW**; values are mean ± SD; **SW:** sedentary women; **AW:** active women; **BMI:** body mass index; **SBP:** systolic blood pressure; **DBP:** diastolic blood pressure; **HR:** heart rate.

No differences were observed regarding perceived stress and mental health aspects of quality of life between groups (Table 2). However, the values found for the AW group indicate a better quality of life in the physical health aspect when compared to the SW group.

**Table 2:** Perceived stress score (PSS‐10) and quality of life in physical and mental health (SF‐36) in the studied groups

Variables	SW (*n* = 48)	AW (*n* = 48)	P
Perceived stress	20.5 ± 6.2	19.9 ± 6.3	0.6392
Physical health	62.1 ± 16.1	68.1 ± 12.6^a^	**0.0448**
Mental health	57.3 ± 18.1	60.1 ± 16.0	0.4240

a Versus. **SW**; Values are mean ± SD; **SW:** sedentary women; **AW:** active women.

**Figure 1:**
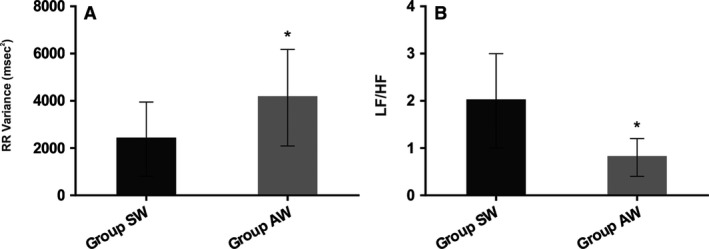
(A) RR variance (msec^2^) at rest in studied groups. (B) Sympatho/vagal balance (LF/HF) at rest in the studied groups. *P < 0.05 versus SW; SW: sedentary women; AW: active women.

For HRV at rest in the time domain, the AW group presented increased RR interval (RR), increased standard deviation of the RR interval (RR SD), increased RR variance and increased RMSSD when compared to the SW group (Table 3, Fig. 1A). In the frequency domain, there was no difference in the absolute values of low frequency (LF); however, significant differences were found in the absolute values of high frequency (HF), percentages of LF and HF (Table 3), and in LF/HF ratio (Fig. 1B).

**Table 3:** Assessment of HRV at rest in the studied groups

Variables	SW (*n* = 48)	AW (*n* = 48)	P
RR (msec)	750.4 ± 76.3	851.5 ± 94.5^a^	**<0.0001**
SD RR (msec)	46.5 ± 14.7	61.7 ± 15.1^a^	**<0.0001**
RMSSD (msec)	32.9 ± 14.3	59.1 ± 25.0^a^	**<0.0001**
Absolute LF (msec^2^)	840.9 ± 443.1	809.3 ± 507.1	0.7458
Absolute HF (msec^2^)	596.0 ± 440.1	1750.9 ± 806.0^a^	**<0.0001**
% LF (n.u.)	57.9 ± 10.6	36.0 ± 13.1^a^	**<0.0001**
% HF (n.u.)	42.1 ± 10.6	64.0 ± 13.1^a^	**<0.0001**

a Versus. SW; values are mean ± SD; SW: sedentary women; AW: active women; RR: RR interval; SD RR: standard deviation of RR interval; RMSSD: Root Mean Square of the Successive Differences; LF: low frequency; HF: high frequency.

**Figure 2:**
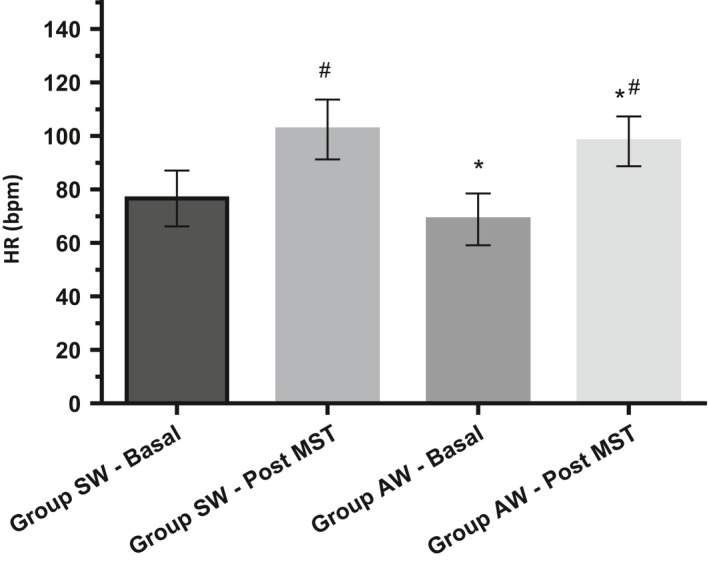
Heart rate (bpm) at rest and immediately after mental stress test (MST). *P < 0.05 versus **SW**; ^#^ *P* < 0.05 versus **Basal**;**SW** **:** sedentary women; **AW** **:** active women.

For heart rate values at rest and after the mental stress test, both groups presented a significant difference (*P* < 0.0001), demonstrating the efficacy of the test (Fig. 2). The results again showed that the AW group maintained a lower HR value after a mentally stressful situation when compared to the SW group (*P* = 0.0348), and the difference was maintained at rest (Table 1).

For time domain measurements of HRV immediately following the mental stress test, the AW group presented increased RR, RR SD, RR variance and RMSSD when compared to the SW group (Table 4). In the frequency domain, there was no difference in the absolute values of LF; however, significant differences were detected in the absolute values of HF, in the percentage of LF and HF, and in the LF/HF ratio (Table 4).

**Table 4:** HRV in the 1st (2–5 min) and 2nd (6–9 min) moments after mental stress test in the studied groups

Variables	SW (*n* = 48)	AW (*n* = 48)	*P*
1st moment after MST			
RR (msec)	757.3 ± 79.4	842.6 ± 89.3^a^	**<0.0001**
SD RR (msec)	51.7 ± 13.9	61.5 ± 18.7^a^	**0.0045**
RR Variance (msec^2^)	2863.4 ± 1403.5	4135.0 ± 2664.3^a^	**0.0043**
RMSSD (msec)	36.2 ± 15.6	57.4 ± 30.9^a^	**<0.0001**
Absolute LF (msec^2^)	1158.6 ± 661.8	971.5 ± 760.8	0.2018
Absolute HF (msec^2^)	701.7 ± 639.3	1701.4 ± 846.3^a^	**<0.0001**
% LF (n.u.)	62.3 ± 11.5	39.0 ± 15.4^a^	**<0.0001**
% HF (n.u.)	37.7 ± 11.5	61.0 ± 15.4^a^	**<0.0001**
LF/HF	2.7 ± 1.3	1.0 ± 0.9^a^	**<0.0001**
2nd moment after MST			
RR (msec)	757.7 ± 82.4	834.3 ± 97.2^a^	**<0.0001**
SD RR (msec)	50.9 ± 15.4	58.7 ± 18.3^a^	**0.0262**
RR Variance (msec^2^)	2827.5 ± 1673.5	3781.0 ± 2262.2^a^	**0.0210**
RMSSD (msec)	36.3 ± 18.1	55.2 ± 31.1^a^	**0.0004**
Absolute LF (msec^2^)	1019.3 ± 767.4	937.3 ± 710.4	0.5882
Absolute HF (msec^2^)	634.9 ± 643.4	1623.6 ± 762.4^a^	**<0.0001**
% LF (n.u.)	62.0 ± 11.7	40.0 ± 14.2^a^	**<0.0001**
% HF (n.u.)	38.0 ± 11.7	60.0 ± 14.2^a^	**<0.0001**
LF/HF	2.4 ± 1.3	0.9 ± 0.6^a^	**<0.0001**

a Versus. SW; values are mean ± SD; MST: mental stress test; SW: sedentary women; AW: active women; RR: RR interval; SD RR: standard deviation of RR interval; RMSSD: Root Mean Square of the Successive Differences; LF: low frequency; HF: high frequency.

For time domain measurements HRV in the 2nd moment after the mental stress test (6–9 min), the AW group showed increased RR, RR SD, RR variance and RMSSD when compared to the SW group (Table 4). In the frequency domain, there was no difference in the absolute values of LF; however, significant differences were observed in the absolute values of HF, in the percentages of LF and HF, and in the LF/HF ratio (Table 4).

For hemodynamic parameters after the recovery period, SBP (SW 106.9 ± 9.3 mmHg vs. AW 108.8 ± 9.7 mmHg) and DBP (SW 68.9 ± 8.3 mmHg vs. AW 69.7 ± 9 mmHg) values were similar between the groups. The AW group (67.6 ± 9.6 bpm) again presented decreased heart rate when compared to SW (76.8 ± 11.6 bpm), *P* < 0.0001.

## Discussion

Although sedentary behavior has been well‐established as one of the main risk factors for cardiovascular disease, few studies have focused on the impact of lifestyle on the modulation of the autonomic nervous system of young women. The present study measured the hemodynamic and autonomic parameters at rest and in response to the mental stress of young sedentary and physically active women, as well as the quality of life in this population. In the study, we observed that sedentary women (SW), without alteration in any conventional clinical parameter, presented impairment in HRV parameters when compared to active ones (AW). In addition, we found that even after a mentally stressful situation, sedentary women still have impairment on parameters of HRV than physically active women. So, the active lifestyle seems to bring autonomic benefits even after a mental stress when compared to sedentary young women.

Over the last few years, lifestyle has become the focus of many studies (Wei et al. 2016; Piepoli and Villani 2017), along with modifiable factors for cardiovascular diseases and their control methods. These studies share an emphasis on the regular practice of physical exercises as a key ally in the prevention and treatment of these diseases.

A study comparing healthy, young, athletic and sedentary male subjects has found that athletes had a reduction in HR when compared to sedentary individuals (Kawaguchi et al. 2007). Grant et al. (2012), in a study involving high‐intensity training program for 12 weeks, have found decreased HR in a group of 183 individuals (100 men and 83 women). Corroborating these studies, our findings indicate that the AW group had a lower HR value than the SW, demonstrating that the regular practice of physical activity was able to slow bradycardia at rest when compared to the sedentary individuals. In this aspect, it should be stressed that the decrease in HR at rest is associated with a good health status, while increased HR is strongly related to mortality risk. This was demonstrated in a cohort study surveying 33,798 people over 22 years, which found that that all‐cause mortality was associated with higher resting HR in men aged 18–59 years and women aged 40–59 years old (Greenland et al. 1999).

In the present study, after resting measurements, we decided to use the Stroop Color Test, since it has been already employed as an effective laboratory stressor (Boutcher and Boutcher 2006), making it possible to induce cardiovascular reactivity and to reproduce a momentary situation of mental stress, which is a contributing factor for morbidity and mortality caused by cardiovascular diseases (Weidner et al. 2001). In this sense, the lower HR of the AW group when compared to the SW group was still maintained after a mental stress situation. This suggests that physical exercise, when performed on a regular basis, is responsible for adaptations in the cardiovascular system both during practice and at rest, enabling the individual to adapt and, as such, to respond more effectively to situations of physiological stress. The bradycardia found in the AW group is possibly related to the increase in cardiac vagal modulation (HF band) and the reduction of the sympathetic modulation (LF band). This was observed in the three stages of analysis of the present study (rest, 1st and 2nd moment after mental stress), both in absolute and in percentage values, when the AW group was compared to the SW group (Camm et al. 1996). This response to mental stress is corroborated by a study (Hamer and Steptoe 2007) associating the highest level of physical fitness with improved responses of HRV variability in 207 subjects of both genders undergoing the Stroop Color Test. Moreover, Gilder et al. (2008) measured seventy‐two women and observed that women reporting higher volumes of physical activity had significantly higher levels of parasympathetic HRV than less active women while in supine; and also demonstrated a much greater change in parasympathetic HRV in response to standing (Gilder and Ramsbottom 2008). This response may well be one of the underlying mechanisms associated with the protection against cardiovascular risk provided by physical conditioning.

In our study, in the analysis of the HRV at rest, in the 1st (2–5 min) and 2nd (6–9 min) moment of recovery after the mental stress test in the time domain, the AW group presented increase in SD RR, RR variance (AW vs. SW) (Fig. 1A) and in the RMSSD when compared to the SW group (Tables 3 and 4). In the frequency domain, there was no difference in the absolute values f low frequency (LF). However, we observed an increase in the high frequency band (HF) in absolute and normalized values, while the normalized values of the band LF (Table 3) and the sympatho/vagal balance (LF/HF: AW vs. SW = *P* < 0.0001) were decreased (Fig. 1B). In line with these findings, a previous study has demonstrated the effect of physical exercise on the cardiovascular response of 34 overweight young undergoing an exercise program assessing the cardiovascular response to the Stroop Color Test. Their findings show that after twelve weeks of the training program, there were significant cardiovascular and autonomic alterations at rest and under mental stress, as decreased HR and increased HRV (Pucci et al. 2012). These positive changes may be related to the increase in high frequency oscillations associated with cardiac vagal modulation. A similar result was found by Middleton and De Vito (Middleton and De Vito 2005), when they compared female athletes with sedentary individuals and found a higher HRV in the athlete group. Furthermore, in a study involving premenopausal and postmenopausal women, it was observed that time and frequency domain parameters of HRV declined with age in healthy sedentary and physically active women. However, the high‐frequency and total power of HRV (variance RR) were higher in the physically active compared with the sedentary women, regardless of age (Davy et al. 1998). In addition, Sandercock et al. (2008) have shown that young men with a higher level of physical activity had lower LF/HF ratio (Fig. 1B), as observed in our findings, since the AW group had a reduction in the sympatho‐vagal balance (LF/HF) when compared to the SW group. This suggests that women who have an active lifestyle through regular physical activity have decreased sympathetic modulation when compared to women who do not exercise regularly. Taken together, our results demonstrated that sedentarism is associated with impairment on HRV parameters in young women.

However, our results show that the benefits of physically active life in women go beyond HR reduction and HRV improvement. The regular practice of physical exercise was also associated with an improvement in the quality of life, which was made clear in the physical health status. A systematic review identified that the highest level of physical activity is associated with a better perception of quality of life in adults (Pucci et al. 2012). Barbosa et al. showed significant gains in the functional capacity, pain and general health domains in the functional group compared to the control group in women (Rezende Barbosa et al. 2016). Thus, the improvement in this aspect for women means a consequent improvement in levels of stress and well‐being, which can positively impact the aging process, while helping in the prevention of chronic noncommunicable diseases.

It should be emphasized that several factors may change the HR in women menstrual cycle. Because of this, all measurements were taken during the follicular phase of the menstrual cycle or in the phase of high hormonal concentration in women who used some type of contraceptive (Rebelo et al. 2012), since this is the phase of greater hormonal stability and HRV.

It should be noted that in assessing physically active women, not athletes, we are addressing the influence of lifestyle rather than high level training, thus enabling such benefits in autonomic modulation to be achieved by anyone who seeks to maintain a proper healthy lifestyle.

In conclusion, it is important to remind that the increased capacity to vary HR is a positive factor of adaptation of the cardiovascular system and consequently of good adaptation of the ANS, and that physical exercise is able to promote functional improvement of the cardiovascular system of healthy individuals and with cardiovascular diseases. Thus, our results demonstrated that sedentary behavior in women induces impairment in autonomic cardiac modulation at rest and in response to physiological stress and worsens their quality of life, even before altering any cardiovascular or metabolic clinical parameters, reinforcing HRV analysis as a possible early marker of cardiovascular risk in this population.

## Conflict of Interest

The authors declare that the results of the study are presented clearly, honestly, and without fabrication, falsification, or inappropriate data manipulation. There is no conflict of interest between authors.
